# Learning to navigate uncertainty in primary care: a scoping literature review

**DOI:** 10.3399/BJGPO.2023.0191

**Published:** 2024-04-03

**Authors:** Nick P Gardner, Gerard J Gormley, Grainne P Kearney

**Affiliations:** 1 Centre for Medical Education, Queen’s University Belfast, Whitla Medical Building, Belfast, Northern Ireland

**Keywords:** uncertainty, education, medical, general practice, primary healthcare

## Abstract

**Background:**

Clinical practice occurs in the context of uncertainty. Primary care is a clinical environment that accepts and works with uncertainty differently from secondary care. Recent literature reviews have contributed to understanding how clinical uncertainty is taught in educational settings and navigated in secondary care, and, to a lesser extent, by experienced GPs. We do not know how medical students and doctors in training learn to navigate uncertainty in primary care.

**Aim:**

To explore what is known about primary care as an opportunity for learning to navigate uncertainty.

**Design & setting:**

Scoping review of articles written in English.

**Method:**

Using a scoping review methodology, Embase, MEDLINE, and Web of Science databases were searched, with additional articles obtained through citation searching. Studies were included in this review if they: (a) were based within populations of medical students and/or doctors in training; and (b) considered clinical uncertainty or ambiguity in primary care or a simulated primary care setting. Study findings were analysed thematically.

**Results:**

Thirty-six studies were included from which the following three major themes were developed: uncertainty contributes to professional identity formation (PIF); adaptive responses; and maladaptive behaviours. Relational and social factors that influence PIF were identified. Adaptive responses included adjusting epistemic expectations and shared decision making (SDM).

**Conclusion:**

Educators can play a key role in helping learners navigate uncertainty through socialisation, discussing primary care epistemology, recognising maladaptive behaviours, and fostering a culture of constructive responses to uncertainty.

## How this fits in

Learning to navigate clinical uncertainty is an essential aspect of medical education. This is the first scoping review to consider what is known about primary care as an opportunity for learning to navigate uncertainty during undergraduate and postgraduate training. The findings suggest that clinical uncertainty influences medical students’ and GP trainees’ professional identity formation (PIF), with both adaptive and maladaptive responses identified. It is important that GP educators are aware of these outcomes when considering the GP training environment and the need for formal teaching about navigating uncertainty.

## Introduction

In clinical practice, physicians make decisions in the context of incomplete information, imperfect cognitive processing, and the potential for unpredictable patient responses. Thus, clinical encounters are always viewed through a lens of uncertainty, with varying degrees of translucency. The ability to navigate uncertainty is an indispensable component of clinical practice and is associated with a spectrum of cognitive, emotional, and behavioural responses.^1^

Uncertainty has been defined as the '*dynamic, subjective perception of not knowing what to think, feel, or do*'.^2^ It has the following two requirements: the absence of understanding; and the presence of conscious awareness.^3^ The extent to which a physician can tolerate uncertainty has recognised influences, including choice of specialisation^4^ and psychological wellbeing.^5^ Patient care is also affected, through timeliness of diagnoses, use of healthcare resources, and prescribing.^6^ GPs with lower tolerance of uncertainty are more likely to refer patients to secondary care colleagues.^7^ These are not benign activities, with potential adverse physical and psychological outcomes for patients and impact on already strained healthcare systems.

Exposure to uncertainty in medical training is necessary for cultivating the professional virtues drawn on when navigating uncertainty.^8^ Individuals are socialised into the culture of their clinical environments, and this includes the extent to which uncertainty is accepted.^9^ Park and Giardino^10^ have described primary care as a *'cultural setting regularly encountering and using uncertainty within consultations*'. Johnston^11^ explained that *'uncertainty and complexity are defining features of the primary care paradigm'*. However, recent literature reviews about navigating uncertainty in training have focused on secondary care settings^12,13^ or undergraduate medical educational interventions.^14^ Alam *et al* ^15^ found that experienced GPs relied on established relationships and heuristics; however, medical students and doctors in training are rarely afforded time in practice to develop these techniques. We propose that individuals learning to practise medicine in primary care may navigate clinical uncertainties differently to experienced clinicians. However, we do not know what is known about primary care as an opportunity for learning to navigate uncertainty in medical training.

## Method

This scoping review was based on the six-step methodological framework developed by Arksey and O’Malley^16^ and advanced by Levac *et al*.^17^ The review was conducted in accordance with the Preferred Reporting Items for Systematic Reviews and Meta-Analysis (PRISMA) extension for scoping reviews.^18^ The researchers in this study included NG, a GP and postgraduate research student; GK, a GP and clinical senior lecturer; and GG, a GP and professor of simulation and clinical skills.

### Identifying the research question

This scoping review sought to answer the question: what is known about primary care as an opportunity for learning to navigate uncertainty? In doing so, its aims were to: (a) identify the extent, range, and nature of the literature available about clinical uncertainty in primary care experienced by medical students and doctors in training; and (b) develop themes that may inform primary care medical educators and influence future research in this area.

### Identifying relevant studies

Studies were included in this review if they: (a) were based within populations of medical students and/or doctors in training; and (b) considered clinical uncertainty or ambiguity in primary care or a simulated primary care setting. Studies based in secondary care, or solely involving senior physicians, were excluded, as were non-English full-text studies.

Search criteria were formulated for three databases: Embase, MEDLINE, and Web of Science, from database inception to present day. Table 1 contains the search terms used. Support was provided by a subject librarian. Although ambiguity’s status as a synonym for uncertainty has generally been adjusted to that of a source or stimulus, it was included in this search as it has been used interchangeably with uncertainty in scientific literature. Search results from each database were imported to the bibliographic management system Covidence.^19^

**Table 1.** Search terms

Primary care	Uncertainty	Education
primary care/primary medical care/primary healthcare general pract* family medic*	uncertain* ambig*	education curricul* training trainee student

### Study selection

**Figure 1.**
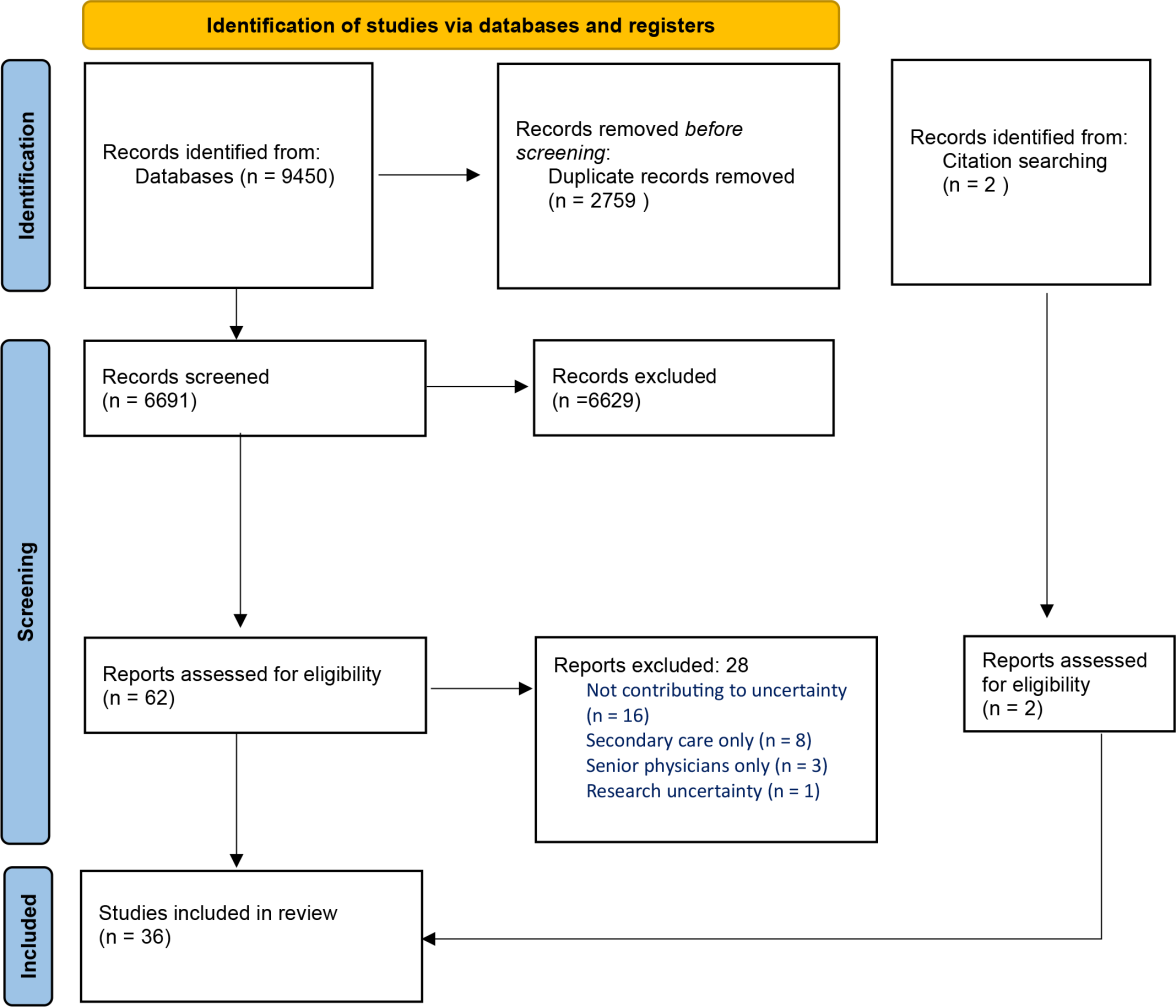
PRISMA flow diagram^60^

Inclusion criteria and search terms were developed in an iterative way during regular research team meetings. The initial screening of studies was conducted by NG. Sixty-two studies met criteria for full-text review, which was conducted by NG and GG independently, with GK arbitrating on studies where NG’s and GG’s decisions differed. All three reviewers were in agreement about the 36 studies included. The reasons for exclusion at this stage are outlined in Figure 1. Citation searching was conducted during the full-text review stage, which yielded two additional studies.

### Charting the data

A data-charting Excel spreadsheet was produced, based on criteria recommended by the JBI Scoping Review Network.^20^ Study findings were analysed thematically, as indicated in Supplementary Table S1. This was an iterative process, with themes initially developed from 10 randomly selected articles with further refinements made throughout. The remaining articles were coded with these themes by one researcher (NG) and discussed as a research team during regular meetings. The team worked reflexively:^21^ shaping themes and discussing issues, ensuring multiple perspectives on the data and rigorous analysis.

## Results

### Search results

Broad keywords used in database searches yielded an initial 6691 studies after duplicates were removed. Once inclusion and exclusion criteria were applied this resulted in 62 studies for full-text review. This yielded 34 studies; two more from reviewing citations brought the total to 36. Study characteristics are shown in Table 2.

**Table 2.** Study characteristics

Characteristic	
Populations	Medical students (12/36) and postgraduate primary care trainees (24/36)
Publication date	Majority after year 2000 (34/36)
Journal affiliations	Majority were family medicine (20) or medical education (10)
Geographical locations	UK (11), Australia (8), US (5), Canada (3), Ireland (2), Germany (2), Norway (2), Sweden (1), France (1), Finland (1)
Study methodologies	Quantitative (15), qualitative (14), mixed-methods (3), opinion pieces (3), one systematic review of 20 studies

### Results of thematic analysis

#### Uncertainty contributes to professional identity formation

Eight^10,22–28^ studies found that clinical uncertainty contributes to professional identity formation (PIF) in primary care. Doctors training in primary care recognised their '*specific role in the broader context of medicine, that when situated with specialists’ narrower expertise*'^23^ allows them to accept uncertainty. Clinical uncertainty, and its impact on trainees’ '*being able to sleep at night*', was found to expedite PIF development as they sought to *'position themselves on a cavalier-to-overcautious spectrum*'.^28^ GP trainees reflected on their professional identity, developing an appreciation of belonging to the wider GP community.^24^ Longitudinal undergraduate primary care placements, where uncertainty contributed to transformed thinking and newfound confidence in the medical student role, were described as *'ideal pedagogic spaces to negotiate the thresholds involved in professional identity formation'*.^26^

PIF is a '*dynamic process achieved through socialisation*',^29^ and 10^23,27,28,30–36^ studies specifically referred to the social role that primary care plays in medical students and GP trainees learning about navigating uncertainty. This relationship-focused strategy^12^ encompasses the role of social resources^37^ pooled from the wider primary care team. GP trainees reported becoming more comfortable with uncertainty by observing senior colleagues’ admissions of uncertainty, appreciating the realisation that '*no-one’s thinking they know it all*'.^28^ This process of socialisation — '*communicative behaviours that contributed to a culture comfort with uncertainty*' — was not information-seeking; its purpose was *'the affective re-appraisal of the role of uncertainty'*.^23^ For medical students, *'identification with more advanced members of "the tribe"'* helped to convert uncertainty into resilience.^27^

#### Adaptive responses

Nine studies^26,28,33–35,38–41^ contributed to understanding the influence that time spent in primary care has on navigating clinical uncertainty. A unanimous finding among these studies was that experience has a positive moderating effect on the discomfort initially associated with navigating uncertainty.

Quantitative analysis^38^ of 594 GP trainees showed that anxiety owing to uncertainty, concern about a bad outcome, and reluctance to disclose diagnostic or treatment uncertainty to patients improves over the duration of training. DeForge and Sobal^40^ found this was independent of the trainees’ age. Medical students undertaking extended placements in primary care developed growing comfort with uncertainty. Over the course of a year their perception of uncertainty changed *'from threatening to challenging to exciting*'.^26^

While experience was independently associated with greater tolerance of uncertainty, additional adaptations to exposure to uncertainty were identified: shared decision making (SDM) and adjusting epistemic expectations. SDM was a finding in nine studies.^10,25,31,42–47^ Often viewed as a '*middle ground'*, SDM is a consultation model found midway between paternalism and informed choice with an emphasis on involving patients in decision making,^44^ and can be considered a '*functional*' adaptation to clinical uncertainty in GP trainees, helping both the doctor and the patient.^31^

Guenter *et a*l^42^ encouraged GP trainees '*to think out loud with the patient about the analytical process*' when navigating uncertainty. Elwyn *et al* ^44^ found that GP trainees recognised that SDM *'unburdens the doctor*'. Several studies in the undergraduate population highlighted the importance of GP tutors verbalising their decision-making processes with medical students. Parekh *et al* ^47^ called for GPs tutors to make the '*implicit explicit'* so that medical students can learn to understand *'the cognitive processes they are working through when seeing patients with … medical uncertainty'*. Park and Giardino^10^ explained that doing so legitimises exploration of uncertainty.

Eleven studies^10,24,26–28,32,35,42,45,48,49^ also identified adjusting epistemic expectations as an adaptation to uncertainty. Han *et al* ^12^ defined this as '*acknowledging the impossibility of perfect medical knowledge and thereby relinquishing the quest for certainty'*. The '*uncertain world of general practice*' creates an environment in which medical knowledge in itself was not an indicator of quality; as a GP trainee explained: *'(You) realise that a lot of these rules and things that we are taught are very black and white, and that’s not true in the context of the person’s life and circumstances …*'^32^ Trainees became comfortable with uncertainty by developing an understanding of the consultation process, by *'readjusting (their) perspective’ to solving problems in general practice over a number of consultations'*.^28^ This also involved adjusting ideas about the role of a competent GP: *'registrars had to leave behind the value that "good" medicine always involved a clear diagnosis and a cure'*.^35^ Medical students considered it a revelation that uncertainty can be accepted as a legitimate professional strategy,^10^ and gained confidence by recognising that *'it’s not always about having a correct answer, it's more the process*'.^26^

#### Maladaptive behaviours

Ten studies^22,31,34,38,42,44,46,50–52^ identified the presence of maladaptive behaviours in medical students and GP trainees. These behaviours took the following two forms: premature closure of diagnostic reasoning associated with actions (admitting, referring, investigating, or prescribing) motivated at imposing certainty too early; and reluctance to disclose uncertainty.^27,31,39,40^

Several studies described GP trainees taking cognitive and behavioural shortcuts in the presence of clinical uncertainty, described as a *'flailing attempt to impose a higher level of certainty on a situation than that situation is ready for*'.^42^ Danczak and Lea^31^ noted that similar management strategies *'seemed to replace analysis with less cognitively demanding, intuitive thinking, leading to action, ending the stress of uncertainty*'. Trainees admitted that they used referrals as *'a way of absolving themselves of uncertainty*',^51^ without considering the negative consequences this could have. Carr and Gormley^46^ also identified this affective stimulus in medical students, whose decisions to investigate patients were *'influenced significantly by the discomfort that they experienced whilst "holding the risk" associated with clinical uncertainty'.* One study^22^ showed greater reluctance to disclose uncertainty to patients was associated with burnout in GP trainees.

### Consultation with stakeholders

When presented in May 2023, the themes of this study resonated with a group of 30 GP educators experienced in training medical students and doctors in training. Moreover, they recognised the maladaptive behaviours described, particularly around excessive investigation and referral, and that socialisation improves this behaviour. GP trainers observed that primary care epistemology changes with experience, and that observing different consultation styles influences GP trainees’ PIF.

## Discussion

### Summary

This scoping review has provided an overview of the existing literature on clinical uncertainty in primary care, as studied in medical students and doctors in training. Thirty-six studies met the inclusion criteria and led to the development of the following three themes observed in both the undergraduate and postgraduate populations: uncertainty contributes to PIF; adaptive responses; and maladaptive behaviours.

### Strengths and limitations

This scoping review is the first to review clinical uncertainty in medical students and doctors in training based in primary care. Methodological strengths include an extensive initial search strategy combined with a review of citations within included studies. However, we may not have included all relevant studies. Although a single-reviewer process was adopted for screening and data extraction, blinded, double screening of full-text reviews and iterative review meetings discussing data charting and thematic analysis added rigour to the methodology. Sharing preliminary findings with stakeholders helped to validate the findings, although consultation was limited to educators. Several included studies did not contribute to any of the themes, and two articles were only available as abstracts.^33,53^

### Comparison with existing literature

Cruess *et al* ^29^ described PIF as influenced by the following three domains: individual (personal characteristics); relational (the influence of a mentor); and collective (the impact of social groups). Findings in this review tended to concentrate on the latter two domains. Medical students and doctors in training valued forming professional relationships in the pursuit of navigating uncertainty, which influenced their professional identity. This was not done exclusively to procure a source of knowledge. Rossignac-Millon and Higgins^54^ explained that '*humans are truth-cartographers searching for epistemic companions with whom to map out the bounds of reality*'. In the same way, medical students and doctors training in primary care form relationships that enable them to better understand the cultural acceptance of uncertainty, and through doing so are socialised into a shared way of thinking and behaving.

Recent frameworks for understanding uncertainty and its tolerance have highlighted moderators of the perception of, and a spectrum of reactions (cognitive, emotional, and behavioural) to, uncertainty.^1,2^ This literature review has correlated with these frameworks, and identified responses in each of these reaction domains that are both adaptive and maladaptive. This review has highlighted PIF, partly through socialisation, as an important moderator of how uncertainty is perceived. The results have also shown an overlap of these ‘response’ domains; for example, the cognitive response of adapting epistemic expectations was inseparable from the emotional responses involved: epistemic adjustments influenced the affective appraisal of uncertainty.

The findings of this review also align with a recent study by Ilgen *et al*.^55^ Studying comfort with uncertainty, the authors have advocated for the need of trainees to adapt their epistemic expectations, calling for educators to *'disabuse their trainees of the notion that clinical knowledge exists in a binary format of "knowing" and "not knowing"*'. They also identified similar maladaptations in response to uncertainty: certainty as a precondition to action, premature diagnostic closure, and overtesting.

Several studies highlighted areas in which medical students and doctors in training were felt to be ill-prepared for managing clinical uncertainty. These included perceptions about the need to *'"fix" the patient'*,^42,51^ and misconceptions about SDM.^44^ As a result, medical students and doctors in training risk experiencing an educational dichotomy between what they are taught and what they experience in practice.^56^

### Implications for research and practice

The results of the study inform several recommendations for undergraduate and postgraduate medical educators. Involving students and trainees in team discussions could provide valuable educational opportunities. Explaining differences in primary and secondary care epistemologies may help in the early navigation of uncertainty. GP educators might benefit from awareness of recognised maladaptive behaviours, and by deliberately fostering a culture of functional responses to uncertainty. Disclosing the degree of uncertainty in the diagnosis should be recommended as an essential aspect of safety netting,^57^ the benefits of SDM within the GP consultation should be emphasised.

It is important to consider whether these recommendations reflect the formal teaching that medical students and GP trainees are receiving. Clinicians and their patients suffer when uncertainty is inexpertly navigated. It is therefore necessary to enquire whether this is reflected in formal undergraduate and postgraduate curricula, considered to be learnt best ‘in-situ’, or simply an overlooked aspect of medical education. This scoping review has highlighted the valuable learning opportunities presented in the everyday exposure to uncertainty in primary care. However, in light of the potential for maladaptive behaviours, is a greater emphasis on evidence-based formal training needed for both GP educators and their learners?

This review has also identified the potential influence that primary care can have on a learner’s epistemological beliefs in the undergraduate and postgraduate populations. Eastwood *et al* ^58^ reported that studies relating to uncertainty have tended to focus on reactions to uncertainty, rather than the nature of epistemic cognition. Future research looking at how uncertainty in primary care influences the nature of epistemic cognition may help inform aspects of medical education programmes.

In conclusion, this scoping review has offered an overview of how medical students and GP trainees navigate clinical uncertainty within primary care. Thematic analysis has identified that uncertainty can be formative in developing professional identity, and that response to it can be adaptive and maladaptive. We have provided concepts that have relevance in helping to shape training in primary care, where uncertainty is a '*constant companion*',^59^ and the only certainty is that there will always be uncertainty.

## Data

All data relied on for the conclusions of this article are available in the supplementary materials.
